# Impact of infection prevention and control on costs, quality, safety and health in long-term care facilities: a systematic review protocol

**DOI:** 10.3389/fpubh.2026.1784637

**Published:** 2026-03-31

**Authors:** Eric Tchouaket, Fatima El-Mousawi, Stephanie Robins, Annie Ménard, Laurence Bernard, Jyoshma Preema D’Souza, Sonia Gull

**Affiliations:** 1 Canada Research Chair in the Economics of Infection Prevention and Control, Department of Nursing, Université du Québec en Outaouais, St-Jérôme Campus, Saint-Jérôme, QC, Canada; 2 Faculty of Nursing, Université de Montréal, Montréal, QC, Canada; 3 Department of Life Sciences and Medicine, Faculty of Science Technology and Medicine, University of Luxembourg, Belval Campus, Esch-sur-Alzette, Luxembourg; 4 University of Luxembourg, Esch-sur-Alzette, Luxembourg

**Keywords:** economic analyses, infection prevention and control, long-term care, protocol, systematic review

## Abstract

**Background:**

Long-term care facilities (LTCFs) are high-risk environments for contracting healthcare-associated infections (HCAIs) due to residents’ vulnerability, close living conditions, and frequent interactions between residents and healthcare staff. HCAIs in LTCFs are preventable through infection prevention and control (IPC) clinical best practices. While prior research has demonstrated the clinical effectiveness of these measures in reducing infection rates, less is known about their cost, impact on quality of life, safety, and health outcomes for residents and healthcare professionals.

**Objective:**

The objective of this systematic review is to assess the impact of IPC measures on costs, quality of life, safety, and health outcomes among residents and healthcare professionals working in LTCFs.

**Methods:**

This systematic review protocol is registered in the Research Registry (reviewregistry1949) and follows the PRISMA 2020 guidelines. The review is grounded in the Institute for Healthcare Improvement framework of infection control interventions and the World Health Organization Infection Prevention and Control Assessment Framework. Eligible studies will include quantitative designs conducted in LTCFs, published between January 1, 2015, and January 1, 2026. Interventions of interest include IPC-related professional (e.g., infection preventionists, IPC nurses) roles and clinical best practices such as hand hygiene, environmental hygiene, screening, and basic and additional precautions. Outcomes will include measures of quality of life, safety, health, and costs. Economic outcomes will be assessed through cost-evaluation studies. Searches will be conducted in CINAHL, MEDLINE, Web of Science, and Cochrane databases. Data extraction will follow CHEERS guidelines for economic studies and STROBE guidelines for non-economic studies. Study quality will be assessed using the Drummond criteria and ROBINS-I guidelines. Monetary values will be standardized to 2025 Canadian dollars, with discounting and sensitivity analyses applied where appropriate.

**Expected results:**

This review will synthesize current evidence on the clinical, economic, and quality-related impacts of IPC measures roles in LTCFs, identifying effective and cost-efficient strategies as well as gaps in existing research.

**Conclusion:**

The findings of this review will provide policymakers, healthcare administrators, and clinicians with evidence-based insights to inform the design and implementation of efficient IPC programs in LTCFs, supporting improved resident outcomes, workforce safety, and sustainable use of healthcare resources.

**Systematic review registration:**

reviewregistry1949.

## Background

Long-term care facilities (LTCFs) are high-risk environments for infectious diseases due to close living in shared spaces, high resident vulnerability, and frequent staff-resident interactions (1). Globally, healthcare-associated infections (HCAIs) affect approximately 3.6% of LTCF residents (1). A longitudinal study across 9 countries in Europe found that 57% of LTCF residents experienced at least one HCAI over 12 months, with an incidence rate of 1.8 HCAIs per 1,000 resident-days (2). Studies have shown that most of these infections could be prevented through the application of infection prevention and control (IPC) measures in LTCFs (3–7). Furthermore, our previous systematic review demonstrated that IPC measures are not only effective in reducing the epidemiologic burden of HCAIs, but also in lowering associated healthcare costs (3). However, neither our previous review, nor other reviews have assessed the impact of IPC measures on the health, security, and quality of life of both residents and healthcare professionals and staff in LTCFs. Therefore, the aim of this systematic review is to assess the impact of IPC measures on costs, quality of life, safety and health of residents and healthcare professionals and staff in LTCFs.

## Methods

### Theoretical framework

This study is grounded in the U. S. Institute for Healthcare Improvement’s (IHI) framework of infection control interventions (8) and is supported by the World Health Organization’s Infection Prevention and Control Core Components Framework, operationalized through the Infection Prevention and Control Assessment Framework (IPCAF) (9). The IHI framework of infection control interventions defines a set of clinical best practices (CBPs) in the form of bundles. The IPCAF provides a structured approach to evaluate and strengthen IPC programs at the facility level, aiming to reduce HCAIs and improve patient safety at the healthcare facility level. The framework emphasizes eight interrelated components that collectively form the foundation for effective IPC activities within a healthcare facility including: (1) IPC programme, (2) IPC guidelines, (3) IPC education and training, (4) HCAI surveillance, (5) multimodal strategies for implementation of IPC interventions, (6) monitoring/audit of IPC practices and feedback, (7) workload, staffing and bed occupancy, and (8) built environment, materials and equipment for IPC at the facility level. Amongst the eight components, the first two directly support the IHI framework of infection control interventions.

### Infection prevention and control, and clinical best practices

For this review, standard IPC measures, which encompass four CBPs will be considered according to the IHI framework of infection control interventions: (1) hand hygiene, (2) hygiene and sanitation of surfaces and equipment, (3) resident screening on admission, and (4) basic and additional precautions (8). These four CBPs were defined in our previous publications (3, 10–12).

### Economic analyses

We will consider five types of economic evaluations: cost-minimization analyses (CMA), cost-effectiveness analyses (CEA), cost-utility analyses (CUA), cost–benefit analyses (CBA) and cost-consequence analyses (CCA). These analyses were described in our previous publications (3, 10–12).

### Research questions and objectives

The main objective of this systematic review is to examine the impact of IPC on LTCFs residents’ quality of life, safety, and health in LTCFs. The systematic review will address the following questions:

1. What are the effects of IPC related healthcare professional roles (e.g., IPC nurses, IPC hygienists, microbiologists, infection preventionists) on residents’ quality of life, safety, and health?
2. What is the impact of IPC CBPs on healthcare professionals’ and residents’ quality of life, safety, and health?
3. What is the economic impact of IPC related professionals’ roles and CBPs on healthcare professionals’ and residents’ quality of life, safety, and health?

### Eligibility criteria

We will include studies that meet the eligibility criteria defined by the Population, Intervention, Comparators and designs, Outcomes and Time (PICOT) framework. These criteria are defined in Table 1.

**Table 1:** Eligibility criteria based on the population, intervention, comparators and designs, outcomes and time (PICOT) framework.

PICOT	Included	Excluded
Population		
Geographic area	All countries	
Establishment	Long-term care: nursing homes, assisted-living facilities, homes for the aged, retirement homes	Independent living communities
Residents and healthcare workers	Residents in long-term care All healthcare professionals (HCP) and staff working in contact with residents Infection preventionists and infection control practitioners	Residents staying <72 h
Interventions Infection prevention and control: Role of HCP Clinical best practices (CBPs)	A) IPC nurses and others specialized in IPC- preventionists and infection control practitioners. B) CBPs: hand hygiene; hygiene and sanitation; screening on admission; basic and additional precautions.	Workplace safety (occupational health officer), dental hygienist
Comparators	No comparators	
Designs	Design: Quantitative studies: controlled clinical trials, randomised clinical trials, cohort studies, longitudinal studies, follow-up studies, prospective studies, retrospective studies, systematic reviews, meta-analyses	Qualitative studies, cross-sectional studies, literature reviews (meta-syntheses, scoping reviews) Technological assessments, purely clinical studies, pharmacological studies, protocols, mathematical/statistical modelling, and simulations
Outcomes	Quality of life, safety, health Cost-effectiveness Net cost savings (savings-costs), incremental cost-effectiveness ratio (ICER = difference of costs/ difference of effectiveness), incremental cost per QALY, incremental cost per disability-adjusted life year (DALY), and incremental benefit–cost ratio (IBCR = savings/costs)	
Timeframe	Jan 1, 2015 to Jan 1, 2026	

### Population (P)

We will restrict this review’s population to LTCF residents and healthcare professionals working in contact with residents. All studies carried out in other healthcare settings (acute care, home care) will be excluded. All countries will be included.

### Intervention (I)

For this review, we will include two types of interventions: (1) roles and actions of IPC nurses and others specialized in IPC (e.g., preventionists and infection control practitioners); and (2) IPC CBPs.

### Comparators and designs (C)

No comparators are defined. We will include quantitative studies including randomised and non-randomised controlled clinical trials, cohort studies, longitudinal studies, follow-up studies, prospective studies, retrospective studies, systematic reviews and meta-analyses. Qualitative studies, cross-sectional studies, literature reviews, mathematical/statistical modelling, and simulations will be excluded.

### Outcomes (O)

Outcomes will include measures of quality of life, safety, health and of economic impact. Economic outcomes will be assessed through cost-effectiveness analyses (including cost-utility, cost-effectiveness, cost–benefit, and cost-consequence) or cost-minimization analyses using the following measures: net cost savings (savings-costs), the incremental cost-effectiveness ratio (ICER = incremental costs/ incremental effectiveness), incremental cost per quality-adjusted life year (QALY), incremental cost per disability-adjusted life year (DALY), and incremental benefit–cost ratio (IBCR = savings/costs).

### Time (T)

Research will include studies published between January 1st, 2015, and January 1st, 2026.

### Information sources

The protocol of this systematic review has been registered in Research Registry under the following unique identifying number: reviewregistry1949. The methods were developed in accordance with the Preferred Reporting Items for Systematic reviews and Meta-Analyses (PRISMA) 2020 statement. A PRISMA checklist (13) is included as Supplementary File 1.

The search will be carried on the following databases: CINAHL, MEDLINE, Web of Science and Cochrane. Database searches will be performed using the Boolean operators “AND” and “OR.” We will only include articles written in English or French and published between January 1st, 2015, and January 1st, 2026.

All co-authors contributed to the determination of keywords. A CINAHL search strategy is presented in Table 2.

**Table 2:** CINAHL search strategy.

Search number	Search string
1	TI (‘*clostridium difficile*’ OR ‘c difficile’ OR ‘c-difficile’ OR ‘c. difficile’ OR ‘c diff’ OR ‘c-diff’ OR ‘c. diff’ OR clostrid* Carbape* OR “hospital acquired” OR “Cross infection” or nosocomial* OR iatrog*) OR AB (‘*clostridium difficile*’ OR ‘c difficile’ OR ‘c-difficile’ OR ‘c. difficile’ OR ‘c diff’ OR ‘c-diff’ OR ‘c. diff’ OR clostrid* OR Carbap* OR “hospital acquired” OR “Cross infection” OR nosocomial* OR iatrog*)
2	TI (‘Urinary-Tract Infections’ OR ‘Urinary-Tract Infection’ OR ‘Blood-Borne Pathogens’ OR ‘acquired pneumonia’ OR pneumonia OR ‘associated pneumonia’ OR flu OR cold) OR AB (‘Urinary-Tract Infections’ OR ‘Urinary-Tract Infection’ OR ‘Blood-Borne Pathogens’ OR ‘acquired pneumonia’ OR ‘acquired pneumonia’ OR ‘associated pneumonia’ OR flu OR cold)
3	(MM “Urinary Tract Infections, Catheter-Related”) OR (MM “Urinary Tract Infections+”)
4	(MM “Bloodborne Pathogens”) OR (MM “Pneumonia, Pneumocystis”) OR (MM “Pneumonia, Viral”) OR (MM “Pneumonia, Aspiration”) OR (MM “Community-Acquired Pneumonia”) OR (MM “Pneumonia, Bacterial+”) OR (MM “Healthcare-Associated Pneumonia”) OR (MM “Pneumonia+”)
5	TI (OR Gastrointestinal OR gastroenteritis) OR AB (Gastrointestinalis OR Gastrointestinal OR gastroenteritis)
6	TI (‘*Haemophilus influenzae*’ OR ‘Respiratory viruses’ OR ‘Influenza viruses’ OR ‘Parainfluenza viruses’ OR Adenoviruses OR ‘*Escherichia coli*’ OR Shigella OR Rotaviruses OR Noroviruses OR Salmonella OR Rhinoviruses OR *Chlamydia pneumoniae* OR Enterovirus) OR AB (‘*Haemophilus influenzae*’ OR ‘Respiratory viruses’ OR ‘Influenza viruses’ OR ‘Parainfluenza viruses’ OR Adenoviruses OR ‘*Escherichia coli*’ OR Shigella OR Rotaviruses OR Noroviruses OR Salmonella OR Rhinoviruses OR *Chlamydia pneumoniae* OR Enterovirus)
7	(MM “Gastroenteritis+”) OR (MM “*Haemophilus Influenzae*”) OR (MM “Haemophilus Infections+”)
8	(MM “Respiratory Syncytial Viruses”) OR (MM “Respiratory Syncytial Virus Infections”) OR (MM “SARS Virus”) OR (MM “*Escherichia Coli*”) OR (MM “*Escherichia Coli* Infections”) OR (MM “Shigella”) OR (MM “Dysentery, Bacillary”) OR (MM “Rotaviruses”) OR (MM “Rotavirus Infections”)
9	(MM “*Chlamydophila Pneumoniae*”) OR (MH “Salmonella Infections”) OR (MM “Caliciviridae Infections”)
10	(MM “Legionella”) OR (MM “Enterovirus Infections+”)
11	TI (Legionella) OR AB (Legionella)
12	TI (‘COVID 19’ OR ‘corona virus’ OR ‘Respiratory syncytial virus’ OR ‘Respiratory infection’ OR ‘Respiratory infections’) OR AB (‘COVID 19’ OR ‘corona virus’ OR ‘Respiratory syncytial virus’ OR ‘Respiratory infection’ OR ‘Respiratory infections’)
13	(MH “Carbapenem-Resistant Enterobacteriaceae”)
14	(MH “Clostridium Infections+”)
15	(MM “Iatrogenic Disease”)
16	(MH “Cross Infection+”)
17	(MM “Tuberculosis, Osteoarticular”) OR (MM “Tuberculosis, Ocular”) OR (MM “Tuberculosis, Gastrointestinal”)
18	TI ((Tuberculosis, Osteoarticular) OR (Tuberculosis, Ocular) OR (Tuberculosis, Gastrointestinal)) OR AB ((Tuberculosis, Osteoarticular) OR (Tuberculosis, Ocular) OR (Tuberculosis, Gastrointestinal))
19	(MM “Scabies”) OR (MM “Skin and Connective Tissue Diseases”) OR (MM “Skin Diseases”) OR (MM “Skin Diseases, Infectious”) OR (MM “Skin Diseases, Parasitic”) OR (MM “Lice Infestations”) OR (MM “Larva Migrans”) OR (MM “Onchocerciasis”) OR (MM “Parasitic Diseases”) OR (MM “Ectoparasitic Infestations”) OR (MM “Mite Infestations”)
20	TI ((Scabies) OR (Skin and Connective Tissue Diseases) OR (Skin Diseases) OR (Skin Diseases, Infectious) OR (Skin Diseases, Parasitic) OR (Lice Infestations) OR (Larva Migrans) OR (Onchocerciasis) OR (Parasitic Diseases) OR (Ectoparasitic Infestations) OR (Mite Infestations)) OR AB ((Scabies) OR (Skin and Connective Tissue Diseases) OR (Skin Diseases) OR (Skin Diseases, Infectious) OR (Skin Diseases, Parasitic) OR (Lice Infestations) OR (Larva Migrans) OR (Onchocerciasis) OR (Parasitic Diseases) OR (Ectoparasitic Infestations) OR (Mite Infestations))
21	(MM “Conjunctivitis”) OR (MM “Conjunctivitis, Acute Hemorrhagic”) OR (MM “Conjunctivitis, Allergic”) OR (MM “Conjunctivitis, Bacterial”) OR (MM “Conjunctivitis, Inclusion”) OR (MM “Conjunctivitis, Viral”)
22	TI ((Conjunctivitis) OR (Conjunctivitis, Acute Hemorrhagic) OR (Conjunctivitis, Allergic) OR (Conjunctivitis, Bacterial) OR (Conjunctivitis, Inclusion) OR (Conjunctivitis, Viral)) OR AB ((Conjunctivitis) OR (Conjunctivitis, Acute Hemorrhagic) OR (Conjunctivitis, Allergic) OR (Conjunctivitis, Bacterial) OR (Conjunctivitis, Inclusion) OR (Conjunctivitis, Viral))
23	#1 OR #2 OR #3 OR #4 OR #5 OR #6 OR #7 OR #8 OR #9 OR #10 OR #11 OR #12 OR #13 OR #14 OR #15 OR #16 OR #17 OR #18 OR #19 OR #20 OR #21 OR #22
24	(TI *Staphylococcus aureus* OR AB *Staphylococcus aureus*) AND (TI methicillin OR AB methicillin)
25	TI (VRE OR ERV) AB (VRE OR ERV)
26	(MH “Methicillin-Resistant *Staphylococcus Aureus*”)
27	(TI Enteroc* OR AB Enteroc*) AND (TI vancomycin OR AB vancomycin)
28	(MH “Vancomycin Resistant Enterococci”)
29	(TI Bacil* OR AB Bacil*) AND (TI Gram OR AB Gram) AND (TI Neg* OR AB Neg*)
30	#24 OR #25 OR #26 OR #27 OR #28 OR #29
31	#23 OR #30
32	TI (Cost* OR econom* OR ‘econom* analysis’ OR efficienc* OR ‘cost effect*’ OR ‘cost util*’ OR ‘cost benefit’ OR ‘cost consequenc*’ OR ‘cost effic*’) OR AB (Cost* OR ‘econom* analysis’ OR econom* OR efficienc* OR ‘cost effect*’ OR ‘cost util*’ OR ‘cost benefit’ OR ‘cost consequenc*’ OR ‘cost effic*’ or ‘cost minimizat*’)
33	(MH “Economics+”)
34	#32 OR #33
35	TI (controlled clinical trial* OR Randomized controlled trial* OR RCT OR blind OR case control* OR Case* OR cohort* OR longitudinal*) OR AB (controlled clinical trial* OR Randomized controlled trial* OR RCT OR blind OR case control* OR Case* OR cohort* OR longitudinal* OR follow up stud* OR prospective stud* OR retrospective stud*)
36	(MH “Randomized Controlled Trials+”) OR (MH “Clinical Trials+”)
37	(MM “Case Studies”) OR (MH “Case Control Studies+”) OR (MH “Matched Case Control”)
38	(MH “Prospective Studies+”)
39	#35 OR #36 OR #37 OR #38
40	TI (Hand* OR Aseptic* OR intervent* OR Program* OR Strateg* OR hygiene* OR Clean* OR control OR prevention OR screen* OR wash OR protect* OR isolation OR sanitation) OR AB (Hand* OR Aseptic* OR intervent* OR Program* OR Strateg* OR hygiene* OR Clean* OR control OR prevention OR screen* OR wash OR protect* OR isolation OR sanitation)
41	(MH “Handwashing+”) OR (MM “Infection Control”) OR (MM “Hygiene”) OR (MH “Patient Isolation+”)
42	TI (“Infection control practitioner” OR “infection prevention and control nurs*” OR (IPC AND nurse) OR “hygiene staff” OR “hygiene and sanitation” OR “microbiologist” OR (hygienist NOT dental)) OR AB (“Infection control practitioner” OR “infection prevention and control nurs*” OR (IPC AND nurse) OR “hygiene staff” OR “hygiene and sanitation” OR “microbiologist” OR (hygienist NOT dental))
43	#40 OR #41 OR #42
44	TI (‘Long-Term Care’ OR ‘Assisted-Living Facilities’ OR ‘long-term-care facility’ OR ‘Homes for the Aged’ OR ‘Nursing Homes’ OR ‘nursing home’ OR ‘long-term care’ OR retirement home) OR AB (‘Long-Term Care’ OR ‘Assisted-Living Facilities’ OR ‘long-term-care facility’ OR ‘Homes for the Aged’ OR ‘Nursing Homes’ OR ‘nursing home’ OR ‘long-term care’)
45	(MM “Outcome Assessment”) OR (MM “Quality of Health Care”) OR (MM “Outcomes (Health Care)”) OR (MM “Medical Futility”) OR (MM “Nursing Outcomes”) OR (MM “Patient-Reported Outcomes”) OR (MM “Treatment Outcomes”) OR (MM “Quality Assurance”) OR (MM “Quality Assessment”) OR (MM “Clinical Documentation Improvement”) OR (MM “Clinical Indicators”) OR (MM “Health Plan Employer Data and Information Set”) OR (MM “Joint Commission Core Measures”) OR (MM “Nursing Audit”) OR (MM “Outcome Assessment Information Set”) OR (MM “Peer Review”) OR (MM “Process Assessment (Health Care)”) OR (MM “Utilization Review”) OR (MM “Program Evaluation”) OR (MM “Accountability”) OR (MM “Guideline Adherence”) OR (MM “Meaningful Use”) OR (MM “Professional Compliance”) OR (MM “Public Reporting of Healthcare Data”) OR (MM “Quality of Nursing Care”) OR (MM “Drug Efficacy”) OR (MM “Fatal Outcome”) OR (MM “Treatment Failure”) OR (MM “Prognosis”) OR (MM “Treatment Termination”) OR (MM “Miscellaneous Techniques”) OR (MM “Global Burden of Disease”) OR (MM “Health Services Research”) OR (MM “Outcomes Research”) OR (MM “Quality of Care Research”) OR (MM “Audit”)
46	(MM “Safety”) OR (MM “Patient Safety”) OR (MM “Accidents”) OR (MM “Decontamination, Hazardous Materials”) OR (MM “Disease Outbreaks”) OR (MM “Disease Transmission”) OR (MM “Drug Contamination”) OR (MM “Environmental Microbiology”) OR (MM “Environmental Pollution”) OR (MM “Exposure to Violence”) OR (MM “Equipment Contamination”) OR (MM “Equipment Reuse”) OR (MM “Fumigation”) OR (MM “Food Safety”) OR (MM “Hygiene”) OR (MM “Infection Control”) OR (MM “Mandatory Reporting”) OR (MM “Mandatory Testing”) OR (MM “Chemical Safety”) OR (MM “Sanitation”) OR (MM “Social Distancing”) OR (MM “Voluntary Reporting”) OR (MM “Occupational Safety”) OR (MM “Equipment Safety”) OR (MM “Fire Safety”) OR (MM “Electrical Safety”) OR (MM “Defibrillators”) OR (MM “Equipment Alarm Systems”) OR (MM “Equipment Design”) OR (MM “Equipment Failure”) OR (MM “Equipment Reliability”) OR (MM “Firefighting Equipment and Supplies”) OR (MM “Incontinence Aids”) OR (MM “Intravenous Therapy Equipment and Supplies”) OR (MM “Lifting and Transfer Equipment”) OR (MM “Hypoallergenic Products”) OR (MM “Safety-Net Providers”) OR (MM “Safety Precautions (Saba CCC)”) OR (MM “Environmental Safety (Saba CCC)”) OR (MM “Equipment Safety (Saba CCC)”) OR (MM “Individual Safety (Saba CCC)”) OR (MM “Violence Control (Saba CCC)”) OR (MM “Safety Component (Saba CCC)”) OR (MM “Catheter Care”) OR (MM “Cultural Safety”) OR (MM “Emergency Care”) OR (MM “Gerontologic Care”) OR (MM “Night Care”) OR (MM “Patient Handling”) OR (MM “Seizure Precautions”) OR (MM “Skin Care”) OR (MM “Wound Care”) OR (MM “Personal Protective Equipment”) OR (MM “Protective Clothing”) OR (MM “Respiratory Protective Devices”) OR (MM “Smoke Alarms”) OR (MM “Ear Protective Devices”) OR (MM “Eye Protective Devices”) OR (MM “Head Protective Devices”) OR (MM “Hip Protectors”) OR (MM “Masks”) OR (MM “Mosquito Nets”) OR (MM “Mouthguards”) OR (MM “United States Occupational Safety and Health Administration”) OR (MM “Emergency Care (Saba CCC)”) OR (MM “Substance Abuse Control (Saba CCC)”) OR (MM “Injury Risk (Saba CCC)”) OR (MM “Violence Risk (Saba CCC)”) OR (MM “Skin Integrity Component (Saba CCC)”)
47	#45 OR #46
48	#31 AND #34 AND #39 AND #43 AND #44 AND #47
	2-15-2026

### Article selection

First, one co-author will query all the databases based on the established search strategies and extract the records. Then, records will be exported to Endnote to create a database of retrieved records where duplicates will be identified and removed. All remaining records will then be exported to the Rayyan platform for screening according to the inclusion and exclusion criteria.

**Figure 1:**
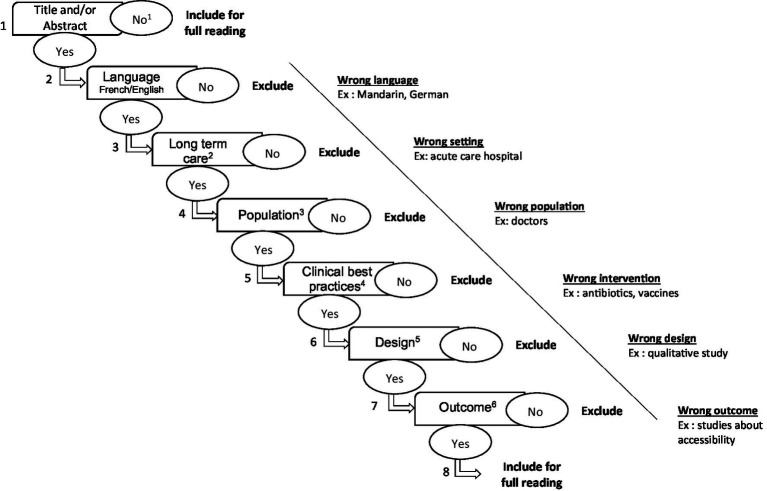
Screening algorithm. ^1^ Reference does or does not have a title and/or abstract. ^2^ Long term care: nursing homes, assisted-living facilities, long-term care facilities, homes for the aged, retirement homes. Excluded: acute care (e.g., hospitals, clinics). ^3^ Population: all residents of long-term care facilities, elder people, IPC, hygienists, infection preventionists, infection control and prevention practionners, médecins infectiologues, médecins microbiologistes, include all community nurses (puis en lisant on vérifie si ces infirmières communautaires sont des personnes dédiées à la PCI ou pas). Excluded: doctors, tout professionnel non dédié à la PCI. ^4^ Clinical best practices: hand hygiene, hygiene and sanitation, screening, basic and additional precautions. Excluded: antibiotics and any other medications. ^5^ Design: quantitative studies (controlled clinical trials, RCTs, cohort studies, longitudinal studies, follow-up studies, prospective studies, retrospective studies, cross-sectional studies). Excluded: qualitative studies, literature reviews (systematic reviews, meta-analyses, meta-syntheses, scoping reviews), studies solely based on mathematical and statistical modelling, protocols. ^6^ Outcome: incremental cost-effectiveness ratio, incremental cost per quality-adjusted life year, incremental cost per disability-adjusted life-year and the incremental cost–benefit ratio, net costs and net cost savings, security, safety. Excluded: studies assessing technology or studies purely clinically oriented.

To improve reliability, prior to the formal selection process, co-authors will independently screen the titles and abstracts of the same 10% subsample of records, followed by a discussion to clarify and agree on the selection process. Three co-authors (FEM, JS, SG) will independently screen all titles and abstracts by following an algorithm developed by the research team (see Figure 1). Records will be included only if the three screening authors are in agreement. In case of conflict, a fourth co-author (SR) will evaluate the title and abstract to arbitrate. Articles corresponding to retained records will undergo full-text review, and those fulfilling the eligibility criteria will be included in the final analysis.

### Data extraction

Data will be extracted from included articles using Microsoft Excel spreadsheets. Two templates will be developed. The first, based on The Consolidated Health Economic Evaluation Reporting Standards (CHEERS) (14) will be used for studies addressing the economic impact of IPC-related professional roles and CBP measures on healthcare professionals’ and residents’ quality of life, safety, and health (third research question).

The second, based on The Strengthening the Reporting of Observational Studies in Epidemiology (STROBE) Statement (15) will be used to extract data from non-economic studies, examining the effects of IPC-related professional roles and the impact of CBP measures on the residents’ quality of life, safety, and health (first and second research questions).

Data extraction will be performed by one co-author (JS) and a generative artificial intelligence chatbot. The articles and spreadsheets will be uploaded to the chatbot with standardized instructions for data extraction. A second co-author (FEM) will compile the data and verify the reliability of the two extractions for each article.

### Assessment of quality of included articles

To ensure the robustness of the results and conclusions of our review, we will assess the quality of the included articles. Economic studies will be assessed using the economic evaluation criteria developed by Drummond et al. (16) while non-economic studies will be assessed using the ROBINS-I guidelines (17). The quality assessment will be conducted independently by two co-authors (FEM and AM) and inter-rater agreement (IRA) will be calculated as a percentage. We will assign a score of 1 in case of agreement and 0 in case of disagreement. Scores will be summed and then divided by the total number of items for each quality assessment tool to obtain the IRA percentage.

To standardize the evaluation and minimize interpretation bias, we will consult a third co-author to agree on statement interpretation of each quality assessment tool before conducting the quality assessment. As in our previous systematic reviews, articles will be classified based on their quality: (1) “high quality,” if the average score across the three tools is at least 80%; (2) “moderate quality,” if the average score is between 60 and 79.9%; and (3) “low quality,” if the average score is less than 60% (3, 10–12).

### Data synthesis

The Dominance Ranking Matrix classification tool will be used to interpret the results of the economic studies included in the review (18). This tool is used to determine whether an intervention should be rejected, favored, or classified as inconclusive.

For all extracted economic data, monetary values reported in difference currencies or price years will be converted to 2025 Canadian dollars (CAD). For economic studies, discount rates of 3, 5 and 8% will be applied. After conversion and discounting, sensitivity analyses will be conducted for economic studies to assess the robustness of monetary results.

For non-economic studies, results will be reported without further analysis. Two types of data will be reported: (1) outcomes and (2) quality of life.

### Ethics and dissemination

This study does not require ethical approval as it will not use individual patient data nor involve human participants, animals or the use of individual patient data. This systematic review is part of the Canada Research Chair in the Economics of Infection Prevention and Control program. The findings will be published in a peer-reviewed journal and presented at scientific conferences.

## Discussion

### Implications

This protocol will result in a review synthesizing the current evidence on the impact of IPC on the quality of life, safety, and health of residents in LTCFs. The findings will provide researchers and policymakers with up-to-date evidence-based information to support more efficient use of limited healthcare resources to ensure the safety and quality of long-term care.

### Limitations and strengths

One potential limitation of this study is the exclusion of qualitative studies, as well as studies examining non-CBP interventions. Despite these limitations, this review will fill an important gap by providing evidence on the impact of IPC-related roles and CBPs on healthcare professionals’ and residents’ quality of life, safety, and health, along with economic outcomes when available. The findings will be supported by a robust assessment process due to the use of quality assessment tools. Another strength of this study is the application of discounting to monetary data, which enables meaningful comparisons across studies in different settings and time periods.
